# Efficacy and safety of tranexamic acid for patients with intertrochanteric fractures treated with intramedullary fixation: A systematic review and meta-analysis of current evidence in randomized controlled trials

**DOI:** 10.3389/fphar.2022.945971

**Published:** 2022-09-19

**Authors:** Jiabao Jiang, Fei Xing, Man Zhe, Rong Luo, Jiawei Xu, Xin Duan, Zhou Xiang

**Affiliations:** ^1^ Department of Orthopedics, Orthopedic Research Institute, West China Hospital, Sichuan University, Chengdu, China; ^2^ Animal Experiment Center, West China Hospital, Sichuan University, Chengdu, Sichuan, China; ^3^ West China School of Medicine, Sichuan University, Chengdu, Sichuan, China

**Keywords:** intertrochanteric fractures, hip fractures, intramedullary fixation, proximal femoral nail autorotation, tranexamic acid

## Abstract

**Background:** Tranexamic acid (TXA) has been widely applied to reduce perioperative bleeding. Recently, several studies focused on the administration of TXA in the treatment for with intertrochanteric fracture patients treated with intramedullary fixation. However, the efficacy and safety of TXA in these studies remain controversial. Therefore, we performed this systematic review and meta-analysis to investigate the efficacy and safety of TXA in intertrochanteric fracture patients treated with intramedullary fixation.

**Methods:** We systematically searched electronic databases, including Cochrane, PubMed, and EMBASE, up to 16 May 2022. The efficacy and safety of TXA was evaluated in four aspects, which were bleeding-related outcomes, non-bleeding-related outcomes, thromboembolic events, and other complications. The outcomes of these studies were extracted and analyzed by RevMan Manager 5.4.

**Results:** Finally, nine randomized controlled trials, involving nine hundred and seventy-two intertrochanteric fracture patients treated with TXA, were enrolled in this study. In the bleeding-related outcomes, TXA group was significantly lower than the control group in terms of total blood loss (MD = −219.42; 95% CI, −299.80 to −139.03; *p* < 0.001), intraoperative blood loss (MD = −36.81; 95% CI, −54.21 to −19.41; *p* < 0.001), hidden blood loss (MD = −189.23; 95% CI, −274.92 to −103.54; *p* < 0.001), and transfusion rate (RR = 0.64; 95% CI, 0.49 to 0.85; *p* = 0.002). Moreover, the postoperative hemoglobin on day 3 of the TXA group was significantly higher than that of the control group (MD = 5.75; 95% CI, 1.26 to 10.23; *p* = 0.01). In the non-bleeding-related outcomes, the length of hospital stays was significantly shorter in the TXA group (MD = −0.67; 95% CI, −1.12 to −0.23; *p* = 0.003). In terms of thromboembolic events, there was no significant differences between the TXA group and control group in deep vein thrombosis, pulmonary embolism, myocardial infarction, and ischemic stroke. As for complications and mortality, there was no significant differences between the TXA group and control group in respiratory infection, renal failure, and postoperative mortality within 1 year.

**Conclusion:** TXA is an effective and safe drug for perioperative bleeding control in intertrochanteric fracture patients treated with intramedullary fixation. However, the long-term efficacy of TXA still needs to be investigated by large-scale multicenter randomized controlled trials.

**Level of evidence:** II, Systematic review and Meta-analysis.

**Systematic Review Registration:** https://inplasy.com/, identifier [INPLASY202280027]

## Introduction

As a common type of low-violence fractures in the elderly, intertrochanteric fracture is mostly related to osteoporosis (Chen et al., 2021). The incidence of intertrochanteric fractures in the elderly increases rapidly. In addition, intertrochanteric fractures account for almost half of all hip fractures (Cheng et al., 2011; Jiang et al., 2022). The number of hip fractures occurring in the world would rise from 1.66 million in 1990 to 6.26 million by 2050, which would place a heavy burden on the healthcare system (Cooper et al., 1992; Milto et al., 2022). For better functional recovery, early surgical treatment is considered the preferred option for treating intertrochanteric fractures (Dehghan and McKee, 2020). In addition, early operation can reduce the occurrence of perioperative complications and postoperative death (Dehghan and McKee, 2020). According to the fracture type, surgical treatments for intertrochanteric fractures can be divided into internal fixation and joint replacement. Intramedullary fixation systems such as proximal femoral nail autorotation (PFNA), are widely used in treating intertrochanteric fractures. Intramedullary fixation systems have advantages of minimally invasive, simple intraoperative operation, reliable fixation strength, and early ambulation (Tang et al., 2012). Perioperative bleeding is an important factor related to the prognosis of hip fracture patients (Ljungqvist et al., 2017). In addition, the intertrochanteric fractures are often accompanied by massive perioperative blood loss, such as intraoperative bleed loss (IBL) and hidden blood loss (HBL) (Foss and Kehlet, 2006; Vochteloo et al., 2011). Currently, various methods have been utilized to decrease the perioperative blood loss in intertrochanteric fracture patients, which includes blood pressure control, autologous blood transfusion, hemostatic drugs, application of tourniquets, and minimally invasive surgery (Carvalho et al., 2015).

Tranexamic acid (TXA) is a synthetic lysine analogue that acts by inhibiting plasminogen activation (Pabinger et al., 2017). In addition, TXA can effectively reduce the amount of bleeding and has been widely used in spine, shoulder, hip, and knee surgery (Hartland et al., 2021; Ling et al., 2021). Recently, more and more researchers applied TXA to reduce perioperative blood loss in the treatment of intertrochanteric fracture patients undergoing intramedullary fixation surgery (Zhou et al., 2019; Yee et al., 2022; Zhang et al., 2022). However, a major limitation of all these trials is the sample size, which may lead to underpowered conclusions. In addition, the efficacy and safety of TXA in intertrochanteric fracture patients treated with intramedullary fixation still remains controversial (Porter et al., 2022). Therefore, we performed this systematic review and meta-analysis to investigate the efficacy and safety of TXA in intertrochanteric fracture patients treated with intramedullary fixation.

## Methods

The Preferred Reporting Items for Systematic reviews and Meta-Analysis (PRISMA) guidelines and Quality of Reporting of Meta-analyses (QUORUM) guidelines were followed in this study (Eriksen and Frandsen, 2018). Our study performed a prospective protocol, consisting of objectives, study selection strategies, inclusion criteria, exclusion criteria, statistical analysis, and outcome measures.

### Search strategy

Two reviewers independently searched for potentially relevant published by using electronic databases, including Cochrane, PubMed, and EMBASE, from inception to 16 May 2022. The following keywords were used for the search: “Tranexamic Acid,” “TXA,” “Hip Fractures,” “Trochanteric Fractures,” “Intertrochanteric Fractures,” “Subtrochanteric Fractures,” “Fragility Fractures,” This strategy was adapted for each included electronic database, and no specific database filters were applied.

### Eligibility criteria

The inclusion criteria for this study were as follows: 1) Population: patients were adults diagnosed with intertrochanteric fractures. The treatment method is intramedullary fixation, include PFNA, Trochanteric femoral nail advanced, Gamma nail, short intramedullary nail, etc. 2) Intervention: patients were treated with TXA. 3) Comparator: patients who received placebo, saline, or blank control. 4) Outcomes: one of the following outcomes was reported. Bleeding-related outcomes consisting of total blood loss (TBL), IBL, HBL, postoperative drainage (POD), blood transfusion rate (BTR), postoperative hemoglobin (Hb) on day 1 or day 3, postoperative hematocrit (Hct) on day 1or day 3. Non-bleeding-related outcomes include the length of hospital stays and surgical time. Thromboembolic events were defined as deep vein thrombosis, pulmonary embolism, myocardial infarction, or ischemic stroke. Other complications include wound complications (wound hematoma or infection), respiratory infection, and renal failure. And postoperative mortality. 5) Study design: the studies were original, randomized control trials (RCTs) only.

The exclusion criteria for this study were as follows: 1) Publications not in the English language. 2) Retrospective studies, cohort studies, case reports, and case series. 3) Animal studies. 4) Nonoriginal research, such as reviews and technical reports. 5) Single abstracts, expert opinions, and letters to editors. 6) Studies in which the relevant data cannot be extracted, and the original author is contacted without a response. Furthermore, any disagreements were resolved by discussion or consulting with another senior reviewer.

### Data extraction

Two reviewers scanned all enrolled studies to extract data independently according to the inclusion and exclusion criteria. The demographic characteristics and surgical information extracted for systematic review were as follows: first author, publication year, country, number of patients, female patients, methods of screening and prophylaxis for thrombosis, the average age of patients, ASA grade, preoperative Hb level, outcome measures, duration of follow-up, fracture types, admission to surgery time, type of anesthesia, surgical procedure, details of the interventions and control, transfusion trigger, postoperative drainage. Means and standard deviations were extracted for outcome measures. We converted medians and interquartile ranges to means and standard deviations for uniform analyses using the methods described by Wan et al. (2014). All data were entered into an electronic spreadsheet. Furthermore, any disagreements were resolved by discussion or consulting with another senior reviewer.

### Assessment of methodological quality

According to Cochrane Collaboration for Systematic Reviews, the methodological quality of trials included in this study was evaluated independently by two reviewers (Corbett et al., 2014). The following items were considered: random sequence generation, allocation sequence concealment, blinding of participants and personnel, blinding of outcomes assessment, incomplete outcome data, selective reporting, and other bias. Each item was assessed as “Low risk of bias,” “Unclear risk of bias,” or “High risk of bias.” If the item was reported incorrectly, the judgment was “High risk of bias.” If the item was reported inadequately, the judgment was “Unclear risk of bias.” If the item was reported correctly and adequately, the judgment was “Low risk of bias.” Furthermore, any disagreements were resolved by discussion or consulting with another senior reviewer.

### Statistical analysis

The statistical analysis was independently performed with RevMan software (Version 5.4; Copenhagen: The Nordic Cochrane Centre, The Cochrane Collaboration, 2020) by two reviewers. The mean difference (MD) between groups of TXA and control was reported with 95% confidence interval (95% CI) and performed to evaluate continuous variables such as TBL. The risk ratio (RR) with 95% CI was performed to evaluate dichotomous outcomes such as BTR. To measure heterogeneity between studies, we used the I^2^ statistic. Furthermore, heterogeneity was accepted, and the randomized-effects model was performed, when I^2^ was>50%. Otherwise, the fixed-effects model was performed. Forest plots were used to graphically represent the difference in outcomes of groups of TXA and control and for all included studies. If *p* values were <0.05, the results were considered statistically significant. Funnel plot was used to assess publication bias. We only performed analyses of partial outcomes. Because funnel plot may not truly reflect possible publication bias when the included studies are few (Higgins and Green, 2011).

## Results

### Included studies

**FIGURE 1:**
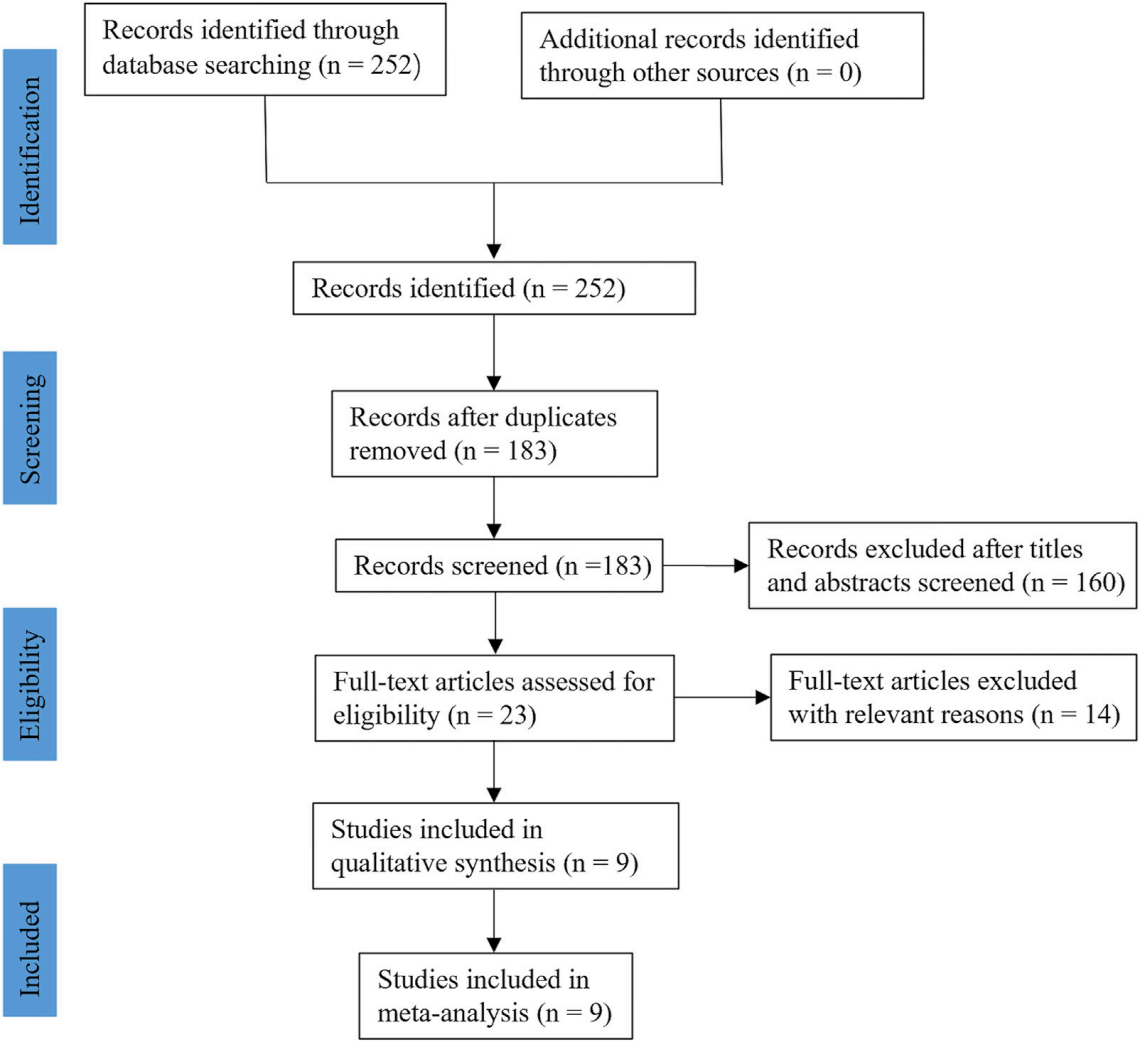
Flow chart of search and search results.

In the initial search, a total of two hundred and fifty-two relevant studies were retrieved. After excluding duplicate studies, one hundred and eighty-three studies were screened using their titles and abstracts, and twenty-three studies were left. Through reading the full text, fourteen studies were excluded because of irrelevant to our topic, no relevant data, non-English publications, and ongoing clinical research that has not yet been published. Finally, nine RCTs (Drakos et al., 2016; Tengberg et al., 2016; Lei et al., 2017; Schiavone et al., 2018; Tian et al., 2018; Luo et al., 2019; Zhou et al., 2019; Yee et al., 2022; Zhang et al., 2022), including nine hundred and seventy-two participants, met the selection criteria and were enrolled in this study. The flow diagram of this study is shown in Figure 1.

### Study characteristics

The main characteristics and surgical information among the nine enrolled RCTs are shown in Table 1 and Table 2, respectively. Among these enrolled studies, six studies were conducted in China (Lei et al., 2017; Tian et al., 2018; Luo et al., 2019; Zhou et al., 2019; Yee et al., 2022; Zhang et al., 2022), each one in Greece (Drakos et al., 2016), Italy (Schiavone et al., 2018) and Denmark (Tengberg et al., 2016). The published years of enrolled RCTs were between 2016 and 2022. Among these included studies, two studies utilized TXA by local administration (Drakos et al., 2016; Yee et al., 2022), and the other studies utilized TXA by intravenous administration (Tengberg et al., 2016; Lei et al., 2017; Schiavone et al., 2018; Tian et al., 2018; Luo et al., 2019; Zhou et al., 2019; Zhang et al., 2022). The fracture type classified by the American Orthopedic/Orthopedic Trauma Association, ranges from 31 A1 to A3. Low molecular weight heparin was used to prevent thromboembolic events in six studies (Drakos et al., 2016; Tengberg et al., 2016; Tian et al., 2018; Luo et al., 2019; Zhou et al., 2019; Zhang et al., 2022). Five studies screened all patients for thromboembolic events such as deep vein thrombosis postoperatively (Lei et al., 2017; Schiavone et al., 2018; Tian et al., 2018; Luo et al., 2019; Zhang et al., 2022), and the other studies performed further imaging and laboratory examination when patients presented with clinical symptoms (Drakos et al., 2016; Tengberg et al., 2016; Zhou et al., 2019; Yee et al., 2022). The procedures for surgery were described in detail in all included studies. Four studies reported that the surgery was performed within 48 h of admission (Drakos et al., 2016; Tengberg et al., 2016; Schiavone et al., 2018; Yee et al., 2022). The implants for intramedullary fixation included PFNA, Trochanteric femoral nail advanced, Gamma nail, and short intramedullary nail. In addition, four studies reported postoperative drainage was performed (Lei et al., 2017; Tian et al., 2018; Zhou et al., 2019; Yee et al., 2022).

**TABLE 1:** The main characteristics of the included studies.

Study (Year)	Country	No. of patients		Female		Age (Year)		ASA grade (3 or 4)		Preop-Hb (g/L)		Thromboprophylaxis	Thrombus screen	Outcome measures	Follow-up (month)
		T	C	T	C	T	C	T	C	T	C						
Drakos et al. 2016	Greece	100	100	73	79	81	80.7	27	20	120	123	LMWH	Clinical	BTR, Hospital stays, Surgical time, Complications, Mortality	12
Lei et al. 2017	China	37	40	32	33	77.80	79.18	12	11	109.5	112.2	NR	Ultrasound	TBL, IBL, HBL, POD, BTR, Postop-Hb on day 1 and day 3, Postop-Hct on day 1 and day 3, Hospital stays, Surgical time, Complications, Mortality	1
Luo et al. 2019	China	44	46	21	26	75.1	76.1	17	20	103	103	LMWH	Ultrasound	TBL, IBL, BTR, Postop-Hb on day 3, Hospital stays, Surgical time, Complications, Mortality	1.5
Schiavone et al. 2018	Italy	47	43	35	28	84.3	84.3	24	23	112	113	NR	Ultrasound	BTR, Complications, Mortality	2
Tengberg et al. 2016	Denmark	33	39	26	25	79.8	75.0	32	38	119.2	128.9	LMWH	Clinical	TBL, IBL, BTR, Complications, Mortality	3
Tian et al., 2018	China	50	50	31	36	77.74	79.25	12	18	NR	NR	LMWH	Ultrasound	TBL, IBL, HBL, POD, BTR, Surgical time, Complications	NR
Yee et al. 2022	China	61	60	40	41	82.2	85.5	NR	NR	110	110	NR	Clinical	TBL, POD, BTR, Surgical time, Complications, Mortality	3
Zhang et al. 2022	China	61	61	33	27	79.11	76.07	53	49	100.07	97.25	LMWH	Ultrasound	TBL, IBL, HBL, BTR, Postop-Hb on day 3, Hospital stays, Surgical time, Complications, Mortality	3
Zhou et al. 2019	China	50	50	35	28	75.1	77.82	13	9	101.60	107.87	LMWH	Clinical	TBL, IBL, HBL, POD, BTR, Postop-Hb on day 1 and day 3, Postop-Hct on day 1 and day 3, Hospital stays, Surgical time, Complications, Mortality	1

T, tranexamic acid group; C, control group; ASA, american society of anesthesiologists; Preop-Hb, Preoperative hemoglobin; Postop-Hb, Postoperative hemoglobin; Postop-Hct, Postoperative hematocrit; LMWH, low molecular weight heparin; TBL, total blood loss; IBL, intraoperative blood loss; HBL, hidden blood loss; POD, postoperative drainage; BTR, blood transfusion rate; NR, not reported.

**TABLE 2:** The surgical information of included studies.

Study (Year)	Fracture types	Admission to surgery time	Anesthesia	Surgical procedure	Intervention	Control	Transfusion trigger	Drainage
Drakos et al. (2016)	AO/OTA 31A1 to 3	≤48 h	Spinal	Closed fracture reduction and fixation with a short cephalomedullary nail (Gamma 3)	Local administration of 3 g TXA at the end of the surgery	No TXA	Hb < 80 g/L or Hct <25%	No
Lei et al. (2017)	AO/OTA 31A1 to 3	NR	NR	Closed fracture reduction and fixation with PFNA	IV 1 g TXA after anesthesia before surgery	Saline	Hb < 90 g/L	Yes
Luo et al. (2019)	AO/OTA 31A1 to 3	NR	Spinal or general	Closed fracture reduction and fixation with PFNA	IV 15 mg/kg TXA 15 min before incision and the same dose again 3 h later	Saline	Hb < 80 g/L	No
Schiavone et al. (2018)	AO/OTA 31A1 to 2	≤48 h	loco-regional or general	Osteosynthesis technique with intramedullary Gamma nail	IV 15 mg/kg TXA at the surgical incision	Saline	Hb < 85 g/L or Hb < 90 g/L for high-risk patients	No
Tengberg et al. (2016)	AO/OTA 31A2.2–31A3	≤24 h	Epidural	Closed fracture reduction and fixation with a short intramedullary nail	IV 1 g TXA before surgery and 3 g TXA at postoperative 24 h	Placebo	Hb < 96.7 g/L	No
Tian et al. (2018)	AO/OTA 31A1 to 3	NR	NR	Closed fracture reduction and fixation with PFNA	IV 10 mg/kg TXA 10 min preoperatively and 5 h postoperatively	No TXA	Hb < 90 g/L	Yes
Yee et al. (2022)	AO/OTA 31 A1 to 3	≤48 h	Spinal or general	Closed fracture reduction and fixation with PFNA or TFNA	Local administration of 1 g TXA following wound closure	Saline	Hb < 80 g/L	Yes
Zhang et al. (2022)	AO/OTA 31 A1 to 3	NR	Spinal or general	Closed fracture reduction and fixation with PFNA	IV 1 g TXA 10 min before incision and 3 h later	Saline	Hb < 70 g/L	No
Zhou et al. (2019)	AO/OTA 31 A1 to 3	≤72 h	Spinal	Closed fracture reduction and fixation with PFNA	IV 1 g TXA 15min before surgery	Placebo	Hb < 70 g/L	Yes

AO/OTA, American Orthopedic/Orthopedic Trauma Association; TXA, tranexamic acid; Hb, hemoglobin; Hct, hematocrit; PFNA, proximal femoral nail autorotation; TFNA, trochanteric femoral nail advanced; IV, intravenous; NR, not reported.

### Quality assessment of individual trials

**FIGURE 2:**
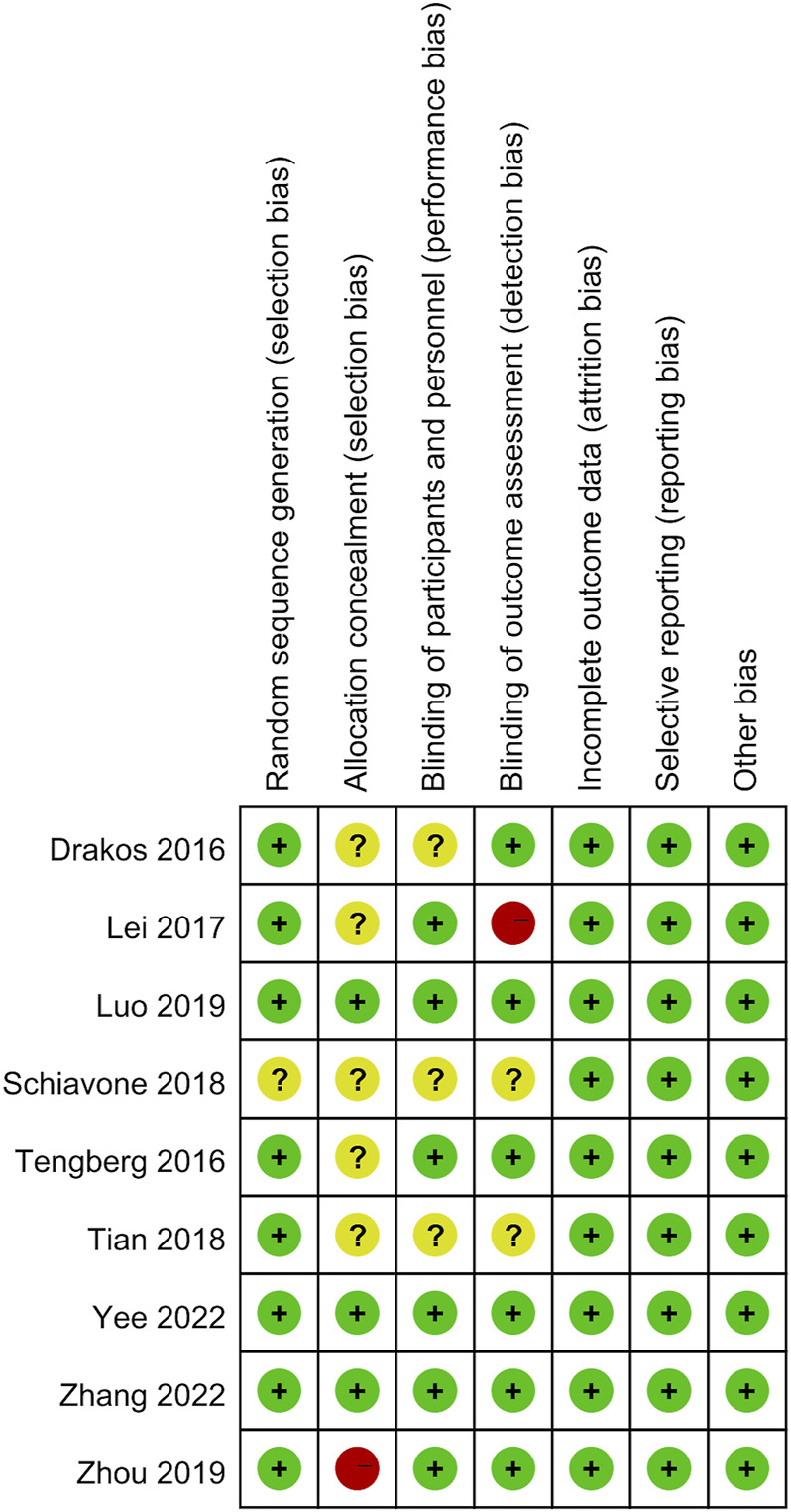
Risk of bias summary: low risk of bias in green; unclear risk of bias in yellow; high risk of bias in red.

**FIGURE 3:**
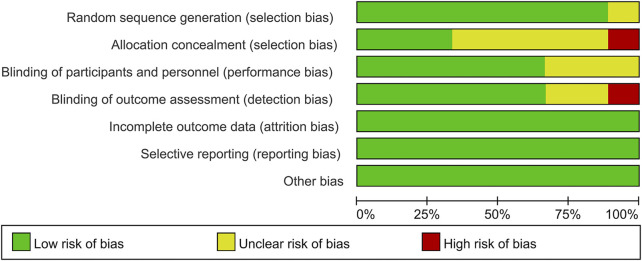
Risk of bias graph: each risk of bias item is presented as the percentage across all the included studies, which indicates the proportion of different levels of risk of bias foreach item.

Among these enrolled studies, eight studies were performed with adequate random sequence generation (Drakos et al., 2016; Tengberg et al., 2016; Lei et al., 2017; Tian et al., 2018; Luo et al., 2019; Zhou et al., 2019; Yee et al., 2022; Zhang et al., 2022). In addition, three studies were conducted with allocation concealment (Luo et al., 2019; Yee et al., 2022; Zhang et al., 2022), and the other studies were not reported and determined to be unclear (Drakos et al., 2016; Tengberg et al., 2016; Lei et al., 2017; Schiavone et al., 2018; Tian et al., 2018; Zhou et al., 2019). Blinding of participants and personnel was conducted in six studies (Tengberg et al., 2016; Lei et al., 2017; Luo et al., 2019; Zhou et al., 2019; Yee et al., 2022; Zhang et al., 2022). Blinding of outcome assessment was reported in six studies (Drakos et al., 2016; Tengberg et al., 2016; Luo et al., 2019; Zhou et al., 2019; Yee et al., 2022; Zhang et al., 2022). In addition, the outcome reports and data of all studies are complete. In addition, no obvious sources of bias in all enrolled trials were identified. Figure 2 shows the risk of bias summary of the included studies. Figure 3 shows the risk of bias graph of the included studies.

### Meta-analysis of bleeding-related outcomes

**FIGURE 4:**
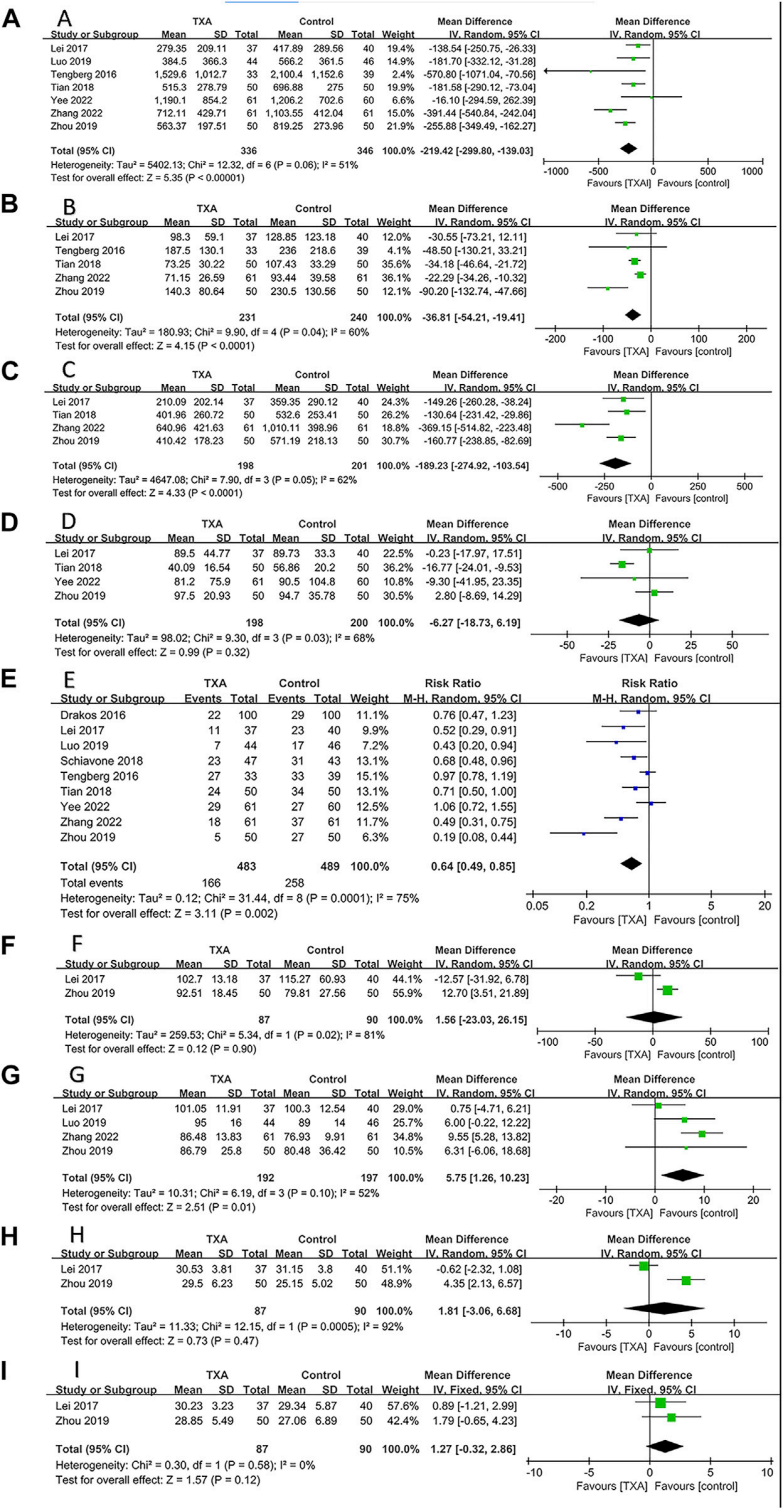
Forest plot showing bleeding-related outcomes of TXA group vs. control group. **(A)** Total blood loss. **(B)** Intraoperative blood loss. **(C)** Hidden blood loss. **(D)** Postoperative drainage. **(E)** Blood transfusion rate. **(F)** Postoperative hemoglobin on day 1. **(G)** Postoperative hemoglobin on day 3. **(H)** Postoperative hematocrit on day 1. **(I)** Postoperative hematocrit on day 3.

All studies reported bleeding-related outcomes, which included four hundred and eighty-three and four hundred and eighty-nine patients in the TXA and control groups, respectively. The results of pooled analyses are shown in Figure 4. The results showed that the TBL of the TXA group was significantly lower than that of the control group (MD = −219.42; 95% CI, −299.80 to −139.03; *p* < 0.001) (Figure 4A). In addition, the IBL and HBL of the PRP group were significantly lower than that of the control group (MD = −36.81; 95% CI, −54.21 to −19.41; *p* < 0.001, MD = −189.23; 95% CI, −274.92 to −103.54; *p* < 0.001) (Figures 4B,C). All enrolled studies reported postoperative BTR. The results showed that the BTR of the TXA group was significantly lower than that of the control group (RR = 0.64; 95% CI, 0.49 to 0.85; *p* = 0.002) (Figure 4E). And the postoperative Hb on day 3 of the TXA group was significantly higher than that of the control group (MD = 5.75; 95% CI, 1.26 to 10.23; *p* = 0.01) (Figure 4G). However, there were no significant differences between TXA group and control group in POD (MD = −6.27; 95% CI, −18.73 to 6.19; *p* = 0.32) (Figure 4D), postoperative Hb on day 1 (MD = 1.56; 95% CI, −23.03 to 26.15; *p* = 0.90) (Figure 4F), postoperative Hct on day 1 (MD = 1.81; 95% CI, −3.06 to 6.68; *p* = 0.47) (Figure 4H), postoperative Hct on day 3 (MD = 1.27; 95% CI, −0.32 to 2.86; *p* = 0.12) (Figure 4I).

### Meta-analysis of non–bleeding-related outcomes

**FIGURE 5:**
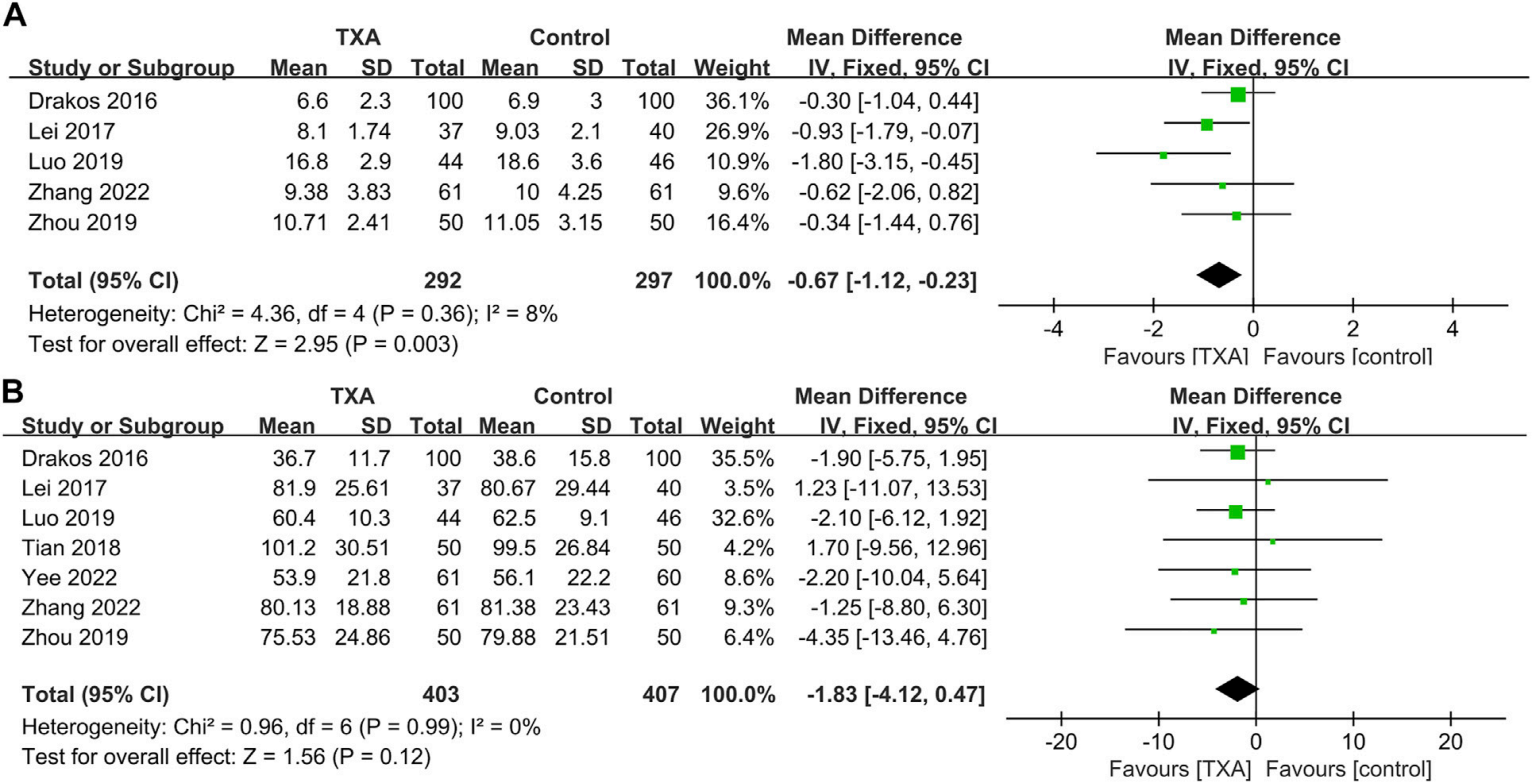
Forest plot showing non–bleeding-related outcomes of TXA group vs. control group. **(A)** Length of hospital stays. **(B)** Surgical time.

Seven studies reported non–bleeding-related outcomes, which included four hundred and three and four hundred and seven patients in the TXA and control groups, respectively. The results of pooled analyses are shown in Figure 5. The results showed that the length of hospital stays of the TXA group were significantly lower than that of the control group (MD = −0.67; 95% CI, −1.12 to −0.23; *p* = 0.003) (Figure 5A). In addition, there were no significant differences between two groups in surgical time (MD = −1.83; 95% CI, −4.12 to 0.47; *p* = 0.12) (Figure 5B).

### Meta-analysis of thromboembolic events

**FIGURE 6:**
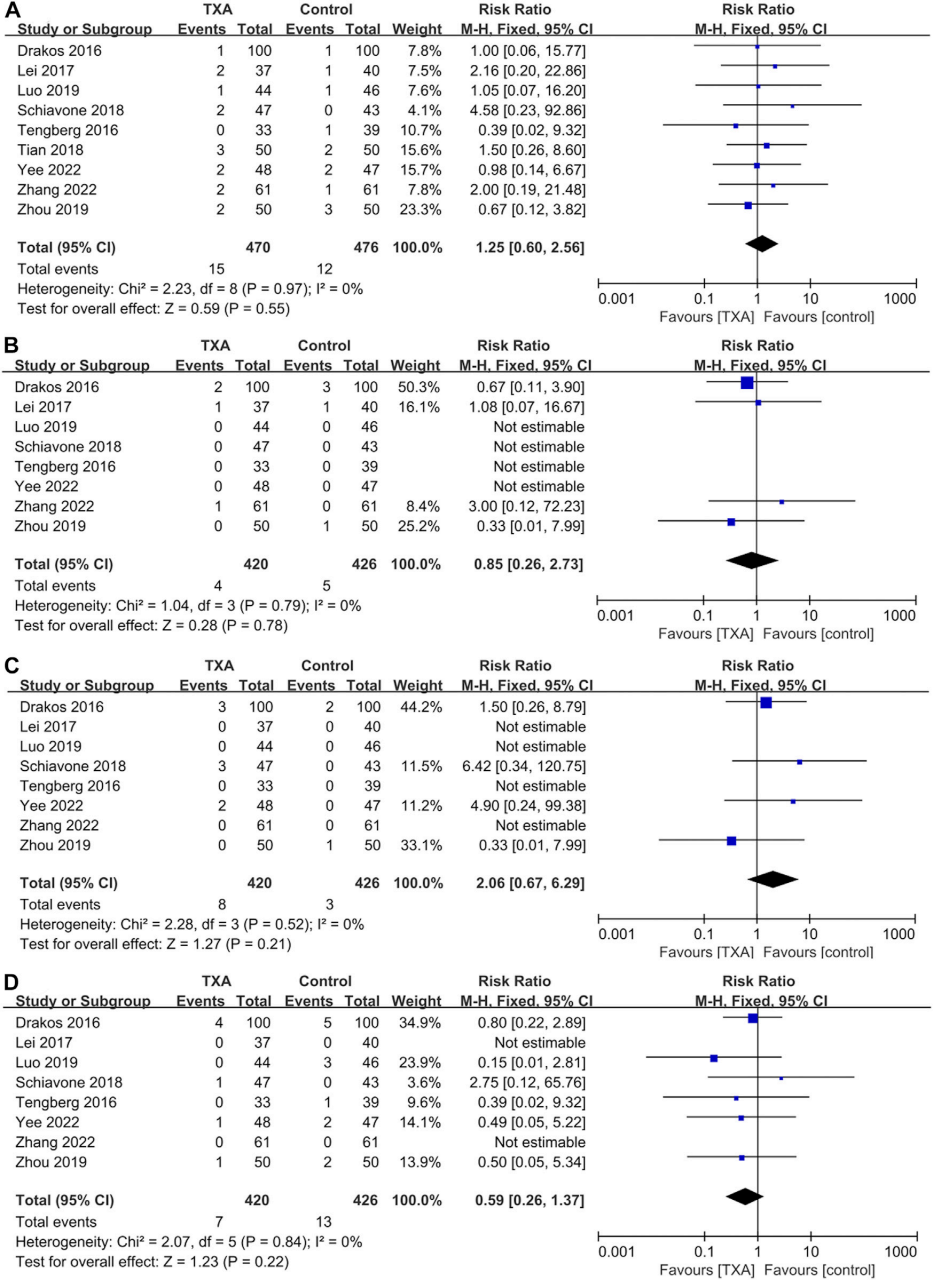
Forest plot showing the incidence of thromboembolic events of TXA group vs. control group. **(A)** Deep vein thrombosis. **(B)** Pulmonary embolism. **(C)** Myocardial infarction. **(D)** Ischemic stroke.

All studies reported thromboembolic events, which included four hundred and seventy and four hundred and seventy-six patients in the TXA and control groups, respectively). The results of pooled analyses are shown in Figure 6. There were no significant differences between two groups in the incidence of deep vein thrombosis (RR = 1.25; 95% CI, 0.60 to 2.56; *p* = 0.55) (Figure 6A), pulmonary embolism (RR = 0.85; 95% CI, 0.26 to 2.73; *p* = 0.78) (Figure 6B), myocardial infarction (RR = 2.06; 95% CI, 0.67 to 6.29; *p* = 0.21) (Figure 6C), ischemic stroke (RR = 0.59; 95% CI, 0.26 to 1.37; *p* = 0.22) (Figure 6D).

### Meta-analysis of other complications

**FIGURE 7:**
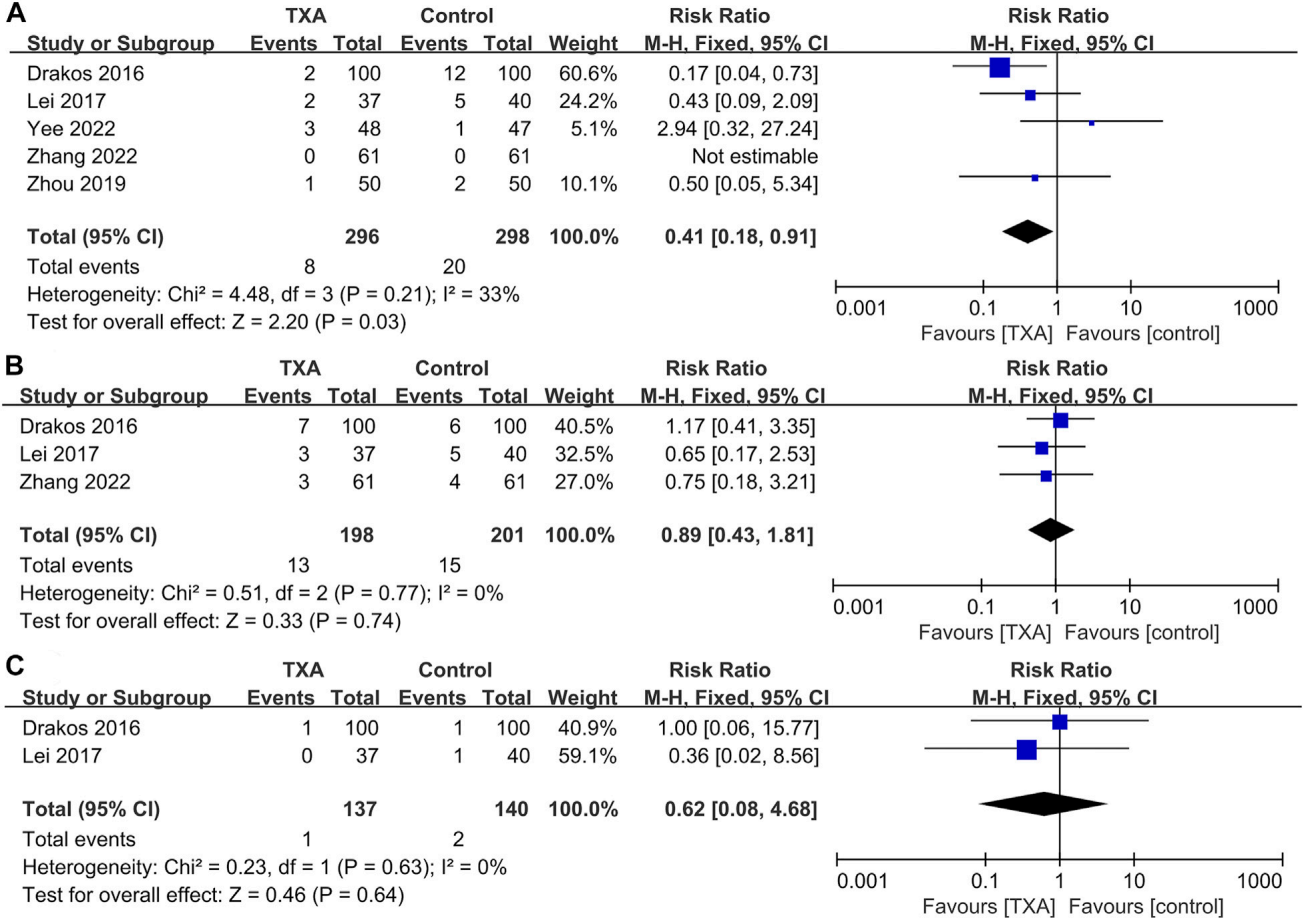
Forest plot showing the incidence of other complications of TXA group vs. control group. **(A)** Wound complications. **(B)** Respiratory infection. **(C)** Renal failure.

Five studies reported complications after surgery, which included two hundred and ninety-six and two hundred and ninety-eight patients in the TXA and control groups, respectively. The results of pooled analyses are shown in Figure 7. The results showed that the incidence of wound complications in the TXA group was significantly lower than that of the control group (RR = 0.41; 95% CI, 0.18 to 0.91; *p* = 0.03) (Figure 7A), And there were no significant differences between the two groups in the incidence of respiratory infections (RR = 0.89; 95% CI, 0.43 to 1.81; *p* = 0.74), renal failure (RR = 0.62; 95% CI, 0.08 to 4.68; *p* = 0.64) (Figures 7B,C).

### Meta-analysis of mortality within 1 year

**FIGURE 8:**
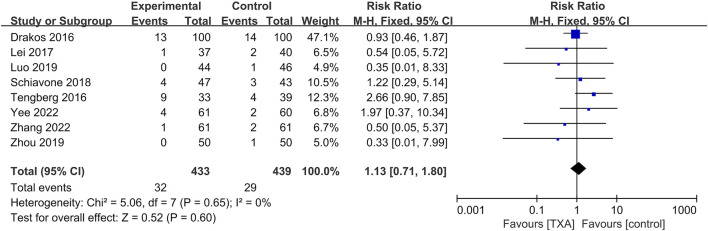
Forest plot showing the mortality within 1 year of TXA group vs. control group.

Eight studies reported postoperative mortality within 1 year, which included four hundred and thirty-three and four hundred and thirty-nine patients in the TXA and control groups, respectively. The results of pooled analyses are shown in Figure 8. The results showed that there were no significant differences between the two groups in postoperative mortality within 1 year (RR = 1.13; 95% CI, 0.71 to 1.80; *p* = 0.60).

### Sensitivity analysis

A sensitivity analysis was performed by individually removing each study to determine whether the pooled results changed. No significant heterogeneity of other outcomes was detected, except for bleeding-related outcomes. As for TBL, I^2^ changed from 51 to 22% after one trial (Zhang et al., 2022) excluded from analysis. I^2^ changed from 60 to 0% after one trial (Zhou et al., 2019) excluded from analysis in terms of IBL. I^2^ changed from 62 to 0% after one trial (Zhang et al., 2022) excluded from analysis in terms of HBL. I^2^ changed from 68 to 0% after one trial (Tian et al., 2018) excluded from analysis in terms of POD. I^2^ changed from 52 to 0% after one trial (Lei et al., 2017) excluded from the analysis in terms of postoperative Hb on day 3. There were no statistically significant changes between groups for all bleeding-related outcomes.

### Publication bias

**FIGURE 9:**
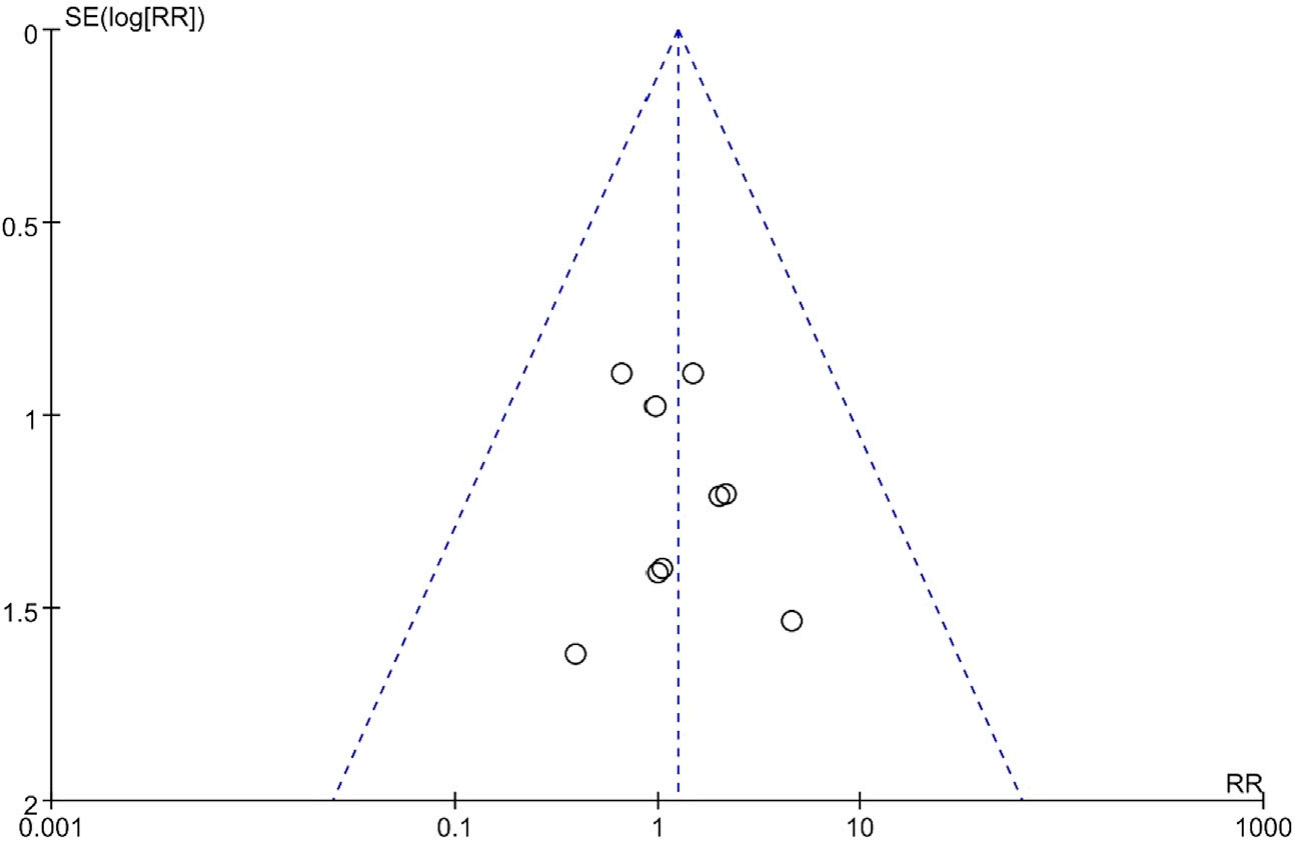
Funnel plot of the incidence of deep vein thrombosis.

The funnel plot of the incidence of deep vein thrombosis showed that the scattered points were roughly symmetrical, suggesting that publication bias among the included studies was mild (Figure 9).

## Discussion

In our study, we conducted a systematic review and meta-analysis to investigate the efficacy and safety of TXA in intertrochanteric fracture patients treated with intramedullary fixation. The results showed that the administration of TXA in intertrochanteric fracture patients treated with intramedullary fixation could significantly reduce TBL, IBL, HBL, and BTR, and significantly increased postoperative HB on day 3. In terms of non-bleeding related outcomes, the TXA group can significantly decrease the length of hospital days compared to the control group. In addition, the administration of TXA would not increase the occurrence of thromboembolic events, respiratory infection, renal failure, and postoperative mortality within 1 year. Furthermore, compared to control group, the administration of TXA can significantly decrease the occurrence of wound complications.

Intertrochanteric fractures are common injuries in the frail geriatric population, and often accompanied with high comorbidities, nutritional deficiencies, and chronic wasting disease, which would increase the occurrence of perioperative anemia (Bross et al., 2010; Strøm Rönnquist et al., 2022). In addition, the intertrochanteric region of the femur is mainly consisted of cancellous bone. Once an intertrochanteric fracture occurs, the amount of blood loss can be very large (Xing et al., 2020a; Xing et al., 2021; Xing et al., 2022). To decrease perioperative blood loss in intertrochanteric fracture patients, intramedullary fixation has been widely used as a minimally invasive surgical treatment. The perioperative TBL of intertrochanteric fracture patients consists of visible blood loss and HBL. In addition, the Sahin et al. demonstrated that the amount of HBL in intertrochanteric fracture patients treated with PFNA accounted for the most of the TBL (Sahin et al., 2014). In addition, perioperative blood management methods, such as hemostatic drugs, tourniquets, and autologous blood recovery, have been applied in surgery of intertrochanteric fracture to reduce intraoperative blood loss (Wong et al., 2021). Allogeneic blood transfusion is another useful method for perioperative control of blood loss. However, allogeneic blood transfusion has disadvantages of viral infection, transfusion reaction, and electrolyte imbalance (Raman et al., 2019).

In recent years, more and more researchers utilized TXA as a promising therapy for perioperative blood control. Transient activation of the fibrinolytic cascade during surgery is one of the reasons for high perioperative blood loss (Banach et al., 2022). TXA can prevent fibrinolysis by blocking the lysine-binding site of plasminogen bound to fibrin and displacing plasminogen from the surface of fibrin (Tanaka et al., 2001). Currently, TXA has been widely used in surgical procedures such as cardiothoracic surgery, neurosurgery, and obstetrics (Yu et al., 2022). In the field of orthopedics, TXA exhibit a high effectiveness in reducing perioperative blood loss in spinal fusion surgery and joint replacement surgery (Ling et al., 2021). However, in the field of trauma orthopedics, the efficacy and safety of TXA in fracture surgery still needs to be further investigated.

Our systematic review and meta-analysis demonstrated that TXA could reduce TBL, IBL, and HBL in intertrochanteric fracture patients treated with intramedullary fixation. Recently, more and more clinicians have recognized the existence of HBL during the hip fracture surgery (Sehat et al., 2000). Previous studies found that HBL can even reach up to 1,473 ml during hip fracture surgery (Foss and Kehlet, 2006). In addition, in the minimally invasive surgery of intertrochanteric fractures, the amount of TBL is also large, and accounts for the vast majority of TBL. Sahin et al. found that the perioperative HBL of PFNA surgery was higher than that of surgery of dynamic condylar screws when dealing with intertrochanteric fractures (Sahin et al., 2014).

The previous study found that TBL was significantly higher in the intramedullary fixation group than in the extramedullary fixation group when dealing with elderly patients with intertrochanteric fractures (Cai et al., 2016). The large amount of HBL in intertrochanteric fractures can be attributed to intraoperative dilation of the medullary cavity, traumatic fibrinolytic hyperlysis, and persistent postoperative bleeding (Smith et al., 2011; Bao et al., 2013). In our study, no significant differences were found between TXA group and control group in terms of POD. BTR is an important factor related with fracture prognosis. Our study found that the administration of TXA can significantly decrease BTR in intertrochanteric fracture patients. The previous study showed that perioperative allogeneic blood transfusions is associated with an increased risk of surgical site infection, urinary tract infection, and overall postoperative infection (Janssen et al., 2015). In addition, the reduced BTR is beneficial in lowing risk of transfusion-related disease and hospitalization costs. In our study, no significant differences were observed in postoperative Hb on day 1 and postoperative Hct on day 1 between the TXA group and control group. The postoperative Hb on day 3 of the TXA group was significantly higher than that of the control group. In our opinions, the higher postoperative Hb of control group might be related to the time-effectiveness of the administration of TXA, which needs further research in future. Based on the results of our study, our study demonstrated that the administration of TXA is effective in reducing blood loss and transfusion rate when dealing with patients with intertrochanteric fractures treated with intramedullary fixation. In terms of non-bleeding-related outcomes, our study showed that the length of hospital stays was significantly shorter in the TXA group compared with the control group. In addition, no significant differences were observed in surgical time between the TXA group and control group. In our opinions, the shorter hospital stays mean the lower patient costs and the lower risk of nosocomial infections.

The incidence of thromboembolic events is an important safe indicator when using TXA in intertrochanteric fracture patients. Whether TXA wound increase the incidence of perioperative thromboembolic events still remains unclear. The previous studies showed that TXA did not increase the risk of thromboembolic events in joint replacement surgery and lumbar fusion (Lu et al., 2018; Ling et al., 2021). However, the activated coagulation system, high age, and various comorbidities of intertrochanteric fracture patients can increase the occurrence of thromboembolic events (Xing et al., 2018). The multicenter RCT based on more than twenty thousand trauma patients demonstrated that the administration of TXA did not increase the risk of thromboembolic events (Shakur et al., 2010). Our study showed that the administration of TXA in patients with intertrochanteric fractures treated with intramedullary fixation did not increase the incidence of deep vein thrombosis, pulmonary embolism, myocardial infarction, and ischemic stroke, which is similar to previous meta-analyses (Xing et al., 2020b; Luo et al., 2020; Li et al., 2021). In our opinions, TXA can reduce blood loss by inactivating the fibrinolytic system rather than by activating the coagulation cascade. However, we also found that some of the included studies did not screen all patients for postoperative thromboembolic events but only after the emergence of relevant clinical symptoms (Drakos et al., 2016; Tengberg et al., 2016; Zhou et al., 2019; Yee et al., 2022). In our opinions, routine postoperative ultrasonography examination is recommended for early detection and diagnosis of thromboembolic events.

Our study also found that the administration of TXA significantly reduced the incidence of wound complications and did not increase the risk of respiratory infection and renal failure. In addition, no significant differences were observed in postoperative mortality within 1 year between TXA group and control group. The main reasons of high mortality rate in the elderly patients with intertrochanteric fractures are various complications such as respiratory infection, lower extremity deep vein thrombosis, and pressure ulcers (Fan et al., 2021). Our study demonstrated that the administration of TXA did not increase the risk of the complications. In addition, among all enrolled studies, four studies conducted surgery within 48 h (Drakos et al., 2016; Tengberg et al., 2016; Schiavone et al., 2018; Yee et al., 2022). The previous study demonstrated that early surgery could reduce the incidence of complications and patient mortality (Pincus et al., 2017). Based on the results of our study, we believe that the administration of TXA is a relatively safe treatment for patients with intertrochanteric fractures treated with intramedullary fixation.

Currently, there is no consensus about the route, dose, and time of administration of TXA in orthopedic surgery (Todeschini et al., 2020). The advantages of topical administration of TXA included maximum concentration at the bleeding site and minimal systemic absorption, thereby reducing the risk of thromboembolic complications and other potential risks (Alshryda et al., 2014). However, another study show no significant differences of local administration of TXA in reducing blood loss and transfusion rates after intertrochanteric fracture surgery (Virani et al., 2016). Dose and time of administration also varied among enrolled studies. Therefore, it is necessary to investigate the optimal route, dose, and time of TXA administrations in the future.

There are several limitations in our study. Firstly, our study is only for patients with intertrochanteric fractures treated with intramedullary fixation, including usual implants such as PFNA and Gamma nails. Secondly, this study included recently published RCTs that reported more outcomes, including bleeding-related outcomes, non-bleeding-related outcomes, thromboembolic events, other complications, and mortality, but the number of included RCTs and the sample size are still small. Thirdly, the follow-up period for the included studies was relatively short. Finally, most of the included studies were conducted in China, and there may be geographical bias.

## Conclusion

This systematic review demonstrated that the administration of TXA was an effective and safe drug for perioperative control of blood loss in patients with intertrochanteric fractures treated with intramedullary fixation. In addition, the administration of TXA did not increase the risk of thromboembolic events such as deep vein thrombosis, pulmonary embolism, myocardial infarction, and ischemic stroke. Moreover, the administration of TXA significantly reduced the incidence of wound complications, with no effect on the incidence of respiratory infection and renal failure, and postoperative mortality within 1 year. However, the long-term adverse effects of TXA need to be further investigated.

## Data Availability

The original contributions presented in the study are included in the article/supplementary material, further inquiries can be directed to the corresponding authors.
